# Supplementary material for: Behavioral differences between infants at and not at elevated risk for autism during a contingency paradigm
Source: PLoS One. 2026 Jan 23;21(1):e0340732. doi: 10.1371/journal.pone.0340732 (PMC12829924; doi:10.1371/journal.pone.0340732)
Supplement: Attachment — Submitted filename: Response to reviewers_BAS_20251215.docx [file pone.0340732.s003.docx]

Response to Editor and Reviewers

Dear Editor,

Thank you for your comments. Below is a response for each point:

- Comment: Please include your tables as part of your main manuscript and remove the individual files. Please note that supplementary tables (should remain/ be uploaded) as separate "Supporting Information" files
  - Response: All tables are now in the main document and have been removed from the submission portal. “Revised Manuscript with Track Changes12_15_2025” is the revised manuscript upload.

Thank you for all your hard work and please let us know if you need anything else from us.

Sincerely,

Marcelo Rosales
